# Impact of Postoperative Chemoradiotherapy versus Chemotherapy Alone on Recurrence and Survival in Patients with Stage II and III Upper Rectal Cancer: A Propensity Score-Matched Analysis

**DOI:** 10.1371/journal.pone.0123657

**Published:** 2015-04-22

**Authors:** Changhoon Song, Sanghyuk Song, Jae-Sung Kim, Heung-Kwon Oh, Duck-Woo Kim, Keun-Wook Lee, Jee Hyun Kim, Keun-Yong Eom, In Ah Kim, Sung-Bum Kang

**Affiliations:** 1 Department of Radiation Oncology, Seoul National University College of Medicine, Seoul National University Bundang Hospital, Seongnam, Korea; 2 Department of Surgery, Seoul National University College of Medicine, Seoul National University Bundang Hospital, Seongnam, Korea; 3 Department of Internal Medicine, Seoul National University College of Medicine, Seoul National University Bundang Hospital, Seongnam, Korea; University of Texas MD Anderson Cancer Center, UNITED STATES

## Abstract

**Purpose:**

To compare the impact of postoperative chemoradiotherapy (CRT) versus adjuvant chemotherapy alone on recurrence and survival in patients with stage II and III upper rectal cancer undergoing curative resection.

**Materials and Methods:**

From our institutional database, 190 patients who underwent primary curative resection between 2003 and 2010 for stage II or III upper rectal cancer were identified. None of the patients received preoperative CRT. Of these, 136 patients received postoperative chemotherapy alone (the CTx group) and 54 patients received postoperative CRT (the CRT group). The CRT group had poorer prognostic features (pT4, pN2, poor differentiation, or involved resection margin) compared with the CTx group. To reduce the impact of treatment selection bias on treatment outcomes, propensity score-matching analysis was used.

**Results:**

The matched cohort consisted of 50 CRT and 50 CTx patients with a median follow-up period of 76 and 63 months, respectively. In the matched cohort, CRT resulted in an improved 5-year local control (98.0% vs. 85.2%, *p* = 0.024) and overall survival rate (89.9% vs. 69.8%, *p* = 0.021) compared with CTx. In the subgroup analysis to identify subpopulations of patients that benefit most from receiving CRT, local recurrence did not occur in patients who did not have poor prognostic features regardless of the receipt of CRT. For patients with any poor prognostic features, CRT resulted in an improved 5-year local control compared with CTx (96.4% vs. 70.7%, *p* = 0.013).

**Conclusions:**

After adjusting for clinicopathologic factors by propensity score-matching, postoperative CRT was associated with improved local control and overall survival in stage II and III upper rectal cancer. Our results suggest that surgery followed by chemotherapy alone is acceptable for patients who did not have poor prognostic features, while additional radiotherapy should be given for patients who have any poor prognostic features.

## Introduction

Preoperative chemoradiotherapy (CRT) has been established as a standard treatment for stage II and III rectal cancer as classified by the American Joint Committee on Cancer (AJCC) [1–4], following demonstration of improved local control and sphincter preservation rates by the German Rectal Cancer Study Group trial [5]. However, the benefit of adjuvant pelvic radiotherapy for upper rectal cancer is not as clear. In the Dutch total mesorectal excision (TME) trial [6] and Swedish rectal cancer trial [7], preoperative short course radiotherapy without chemotherapy conferred no significant benefit on local control for tumors located 10.1 to 15.0 cm from the anal verge. This result suggests adjuvant pelvic radiotherapy for upper rectal cancer is ineffective. Another concern with postoperative adjuvant pelvic radiotherapy for upper rectal cancer is that the upper rectum is covered by peritoneum anteriorly and on both sides, while the mid rectum is only covered anteriorly and there is no peritoneal covering in the lower rectum. Consequently, for a given transmural invasion depth of primary tumors, upper rectal tumors are more likely to penetrate the peritoneum than lower rectal tumors. Tumors penetrating the peritoneum are deemed more likely to disseminate within the peritoneal cavity and be beyond the field of pelvic radiotherapy [8]. For this reason, the current treatment guidelines for AJCC stage II or III upper rectal cancer are imprecise. The National Comprehensive Cancer Network (NCCN) guidelines recommend preoperative CRT regardless of the location of tumor [4]. However, the American Society of Colon & Rectal Surgeons (ASCRS) recommends either preoperative or postoperative CRT for upper rectal cancer [9], while the German S3 guideline recommends either adjuvant chemotherapy as in the guidelines for colon cancer or perioperative radiotherapy with or without chemotherapy as in the guidelines for rectal cancer [2]. Different treatment strategies have been used in our institute according to the location of the tumor. For mid and lower rectal cancers clinically staged as II and III, preoperative CRT followed by surgery and adjuvant chemotherapy is preferred [10, 11]. In contrast, for upper rectal cancers, most patients undergo surgery followed by adjuvant chemotherapy, while postoperative CRT is only considered in select patients.

Although several studies have reported surgical outcomes of upper rectal cancer, the role of adjuvant CRT has not been evaluated [12–15]. Therefore, we evaluated the impact of postoperative CRT on recurrence and survival in patients with stage II and III upper rectal cancer.

## Materials and Methods

### Patients and pretreatment evaluation

This retrospective study, based on the data from the registry of colorectal cancer which was prospectively collected and maintained in our hospital, was approved by the Institutional Review Board of Seoul National University Bundang Hospital. The IRB waived the written informed consent from patients due to the retrospective nature of this study. Between July 2003 and December 2010, 685 patients underwent surgery for rectal cancer at our hospital. Patients with mid and lower rectal cancer (n = 428), clinical/pathological evidences of distant metastases at initial presentation (n = 43), another malignancy (n = 11), recurrent cancer (n = 1), tumor stage of I (n = 2), palliative resection (n = 1), and hereditary nonpolyposis colorectal cancer (n = 1) were excluded. As we aimed to analyze the impact of postoperative CRT on recurrence and survival, patients who died within 30 days of surgery or any time during the original hospital stay regardless of duration (n = 2) and those patients with no disease recurrence who were followed-up for < 12 months (n = 6) were also excluded. Ultimately, a total of 190 patients with stage II and III primary adenocarcinoma of upper rectum were included in this study. As per institutional policy, all upper rectal cancers undergo surgery directly, without preoperative treatment. Therefore, none of upper rectal cancer patients in our hospital received preoperative CRT.

All patients with rectal cancer were assessed by either of two experienced colorectal surgeons (SBK and DWK) during their first visit. The lower margin of the tumor from the anal verge was assessed by preoperative digital rectal examination (DRE), magnetic resonance imaging (MRI), computed tomography (CT), colonoscopy and/or rigid proctoscopy. The upper rectal cancer was defined preoperatively as the inferior margin of the tumor between 9 and 15 cm from the anal verge by DRE, image, and endoscopic study and the tumor being not palpable on DRE. Partially peritonealized tumor location was also identified on the operative note and pathologic report. The tumor stage was classified by using the 7th edition of the AJCC staging system. In all patients, staging workup included DRE, complete blood count, liver/renal function tests, carcinoembryonic antigen (CEA) measurement, colonoscopy, CT of the abdominopelvic region or MRI of the pelvis, and chest radiography. If distant metastasis was suspected, further evaluations such as MRI of the liver, positron emission tomography, or chest CT were performed. Involved resection margins were defined as tumor cell involvement within 2 mm of the circumferential resection margin or within 5 mm of the distal resection margin [16–20].

### Treatment

Either of two experienced colorectal surgeons (SBK and DWK) performed all operations. A tumor-specific mesorectal excision was used as per the ASCRS practice parameters for the treatment of rectal cancer [14, 21]. Postoperative chemotherapy for 6 months was recommended for all patients. Postoperative concurrent CRT was administered to 54 patients (the CRT group), while the remaining 136 patients (the CTx group) only received postoperative chemotherapy. The decision on whether or not to administer postoperative radiotherapy in addition to postoperative chemotherapy was at the discretion of the surgeon (SBK and DWK) or radiation oncologist (JSK). The technical aspects of postoperative radiotherapy have been described previously [11]. All radiation therapy was performed by a single experienced radiation oncologist (JSK). The pelvis was irradiated with a dose of 45 Gy followed by a primary tumor bed boost of 5.4 Gy, except in four patients. In two patients, no boost was administered because a large volume of the small bowel was included in the radiation field. In two other patients, a 10.8 Gy boost was administered because of an involved resection margin. All patients who received radiotherapy underwent concurrent chemotherapy. The most common chemotherapy regimens during radiotherapy were 2 cycles of an intravenous bolus injection of fluorouracil (5-FU) plus leucovorin (LV) for 3 days in the first and fifth weeks of radiotherapy or continuous oral administration of capecitabine throughout radiotherapy. The regimens for postoperative adjuvant chemotherapy were oral capecitabine, 5-FU plus LV (FL), FL plus oxaliplatin (FOLFOX), or oral tegafur/uracil plus LV.

### Statistical analysis

The statistical significance of differences was assessed using the Chi squared test or Fisher’s exact test for categorical data, and the t-test or Mann–Whitney U test, for continuous data. Overall survival (OS) was defined as the time from the date of surgery to the date of death from any cause. Relapse-free survival (RFS) was calculated as the time from the date of surgery to the detection of recurrent disease or death, whichever occurred first. The local control rate was defined as the time from the date of surgery to the date of relapse detected in pelvic cavity. Distant-metastasis free (DMF) rate was calculated as the time from the date of surgery to the detection of distant metastasis. Survival curves were generated using the Kaplan-Meier method, and a univariate survival comparison was performed using the log-rank test.

Since patients were not randomly assigned to receive postoperative chemotherapy alone (the CTx group) or postoperative CRT (the CRT group), it was highly likely that the two patients groups would have significant baseline differences that could confound the analysis of final outcomes. In order to reduce the effect of treatment-selection bias and simulate the effects of randomization, propensity score-matching was used [22]. Propensity scores were estimated using a logistic regression model based on age, sex, pT stage, pN stage, histologic grade, involved resection margin, and adjuvant chemotherapy. One-to-one matching without replacement was performed using a 0.2 caliper width and the resulting score-matched pairs were used in subsequent analyses as indicated.

All analyses were carried out with statistical program R (R Foundation for Statistical Computing, Vienna, Austria. http://www.R-project.org). All *p* values reported are two-sided, with *p* < 0.05 used to denote statistical significance.

## Results

Descriptive statistics relating to patient, tumor, and treatment characteristics for the entire cohort (n = 190), as well as the propensity score-matched cohort (n = 100), are summarized in Table 1.

**Table 1:** Characteristics of all patients (n = 190) and patients matched on propensity scores (n = 100), stratified by treatment type (CTx vs CRT).

Variable		Entire cohort (n = 190)			Propensity score-matched cohort (n = 100)		
		CTx (n = 136)	CRT (n = 54)	*P* value	CTx (n = 50)	CRT (n = 50)	*P* value
**Age**				0.057			0.546
	**≤ 60 years**	50 (36.8)	28 (51.9)		21 (42.0)	24 (48.0)	
	**> 60 years**	86 (63.2)	26 (48.1)		29 (58.0)	26 (52.0)	
**Sex**				0.066			0.822
	**Female**	56 (41.2)	14 (25.9)		14 (28.0)	13 (26.0)	
	**Male**	80 (58.8)	40 (74.1)		36 (72.0)	37 (74.0)	
**pT stage**				0.782			1.000
	**pT1/2**	13 (9.6)	4 (7.4)		4 (8.0)	4 (8.0)	
	**pT3/4**	123 (90.4)	50 (92.6)		46 (92.0)	46 (92.0)	
**pN stage**				0.023*			0.425
	**pN0**	64 (47.1)	16 (29.6)		19 (38.0)	15 (30.0)	
	**pN1**	47 (34.6)	19 (35.2)		13 (26.0)	19 (38.0)	
	**pN2**	25 (18.4)	19 (35.2)		18 (36.0)	16 (32.0)	
**pTNM stage**				0.028*			0.398
	**pStage II**	64 (47.1)	16 (29.6)		19 (38.0)	15 (30.0)	
	**pStage III**	72 (52.9)	38 (70.4)		31 (62.0)	35 (70.0)	
**Histologic grade**				0.197			1.000
	**Others**	130 (95.6)	49 (90.7)		45 (90.0)	46 (92.0)	
	**Poorly differentiated**	6 (4.4)	5 (9.3)		5 (10.0)	4 (8.0)	
**Tumor size (cm)**		4.8 (1.0–12.0)	5.0 (0.2–9.0)	0.689	5.0 (1.0–10.0)	5.0 (0.2–9.0)	0.922
**Number of harvested lymph nodes**		27 (7–119)	26 (12–76)	0.087	28 (7–119)	25 (12–65)	0.059
**Preoperative CEA (ng/mL)**		2.5 (0.9–215.0)	2.1 (0.9–40.0)	0.382	2.7 (0.9–122.3)	2.1 (0.9–40.0)	0.325
**Postoperative CEA (ng/mL)**		1.1 (0.3–11.2)	1.1 (0.5–4.5)	0.391	1.1 (0.8–11.2)	1.1 (0.5–4.5)	0.426
**Lymphovascular invasion**				0.031*			0.839
	**No**	74 (54.4)	20 (37.0)		21 (42.0)	20 (40.0)	
	**Yes**	62 (45.6)	34 (63.0)		29 (58.0)	30 (60.0)	
**Perineural invasion**				0.976			0.391
	**No**	96 (70.6)	38 (70.4)		32 (64.0)	36 (72.0)	
	**Yes**	40 (29.4)	16 (29.4)		18 (36.0)	14 (28.0)	
**Involved resection margin**				<0.001*			0.183
	**No**	128 (94.1)	40 (74.1)		44 (88.0)	39 (78.0)	
	**Yes**	8 (5.9)	14 (25.9)		6 (12.0)	11 (22.0)	
**Adjuvant chemotherapy for 6 months**				0.027*			1.000
	**No**	22 (16.2)	2 (3.7)		2 (4.0)	2 (4.0)	
	**Yes**	114 (83.8)	52 (96.3)		48 (96.0)	48 (96.0)	

Categorical variables are presented as n (%), continuous variables are presented as median (range).

*Abbreviations*: CTx = chemotherapy alone; CRT = chemoradiotherapy; CEA = carcinoembryonic antigen.

* Statistically significant.

### Entire cohort prior to propensity score matching

In the entire cohort, CRT group patients had poorer prognostic features than CTx group patients. There was a significantly higher proportion of patients with pN2 stage (35.2% vs. 18.4%), pStage III (70.4% vs. 52.9%), lymphovascular invasion (63.0% vs. 45.6%), and involved resection margin (25.9% vs. 5.9%) in the CRT group than in the CTx group. The number of harvested lymph nodes and the proportion of females were lower in the CRT group than in the CTx group, with a trend toward significance (*p* = 0.087 and *p* = 0.066, respectively). In contrast, the proportion of younger patients was higher in the CRT group than in the CTx group (*p* = 0.057). Finally, the proportion of patients who received 6 months of adjuvant chemotherapy as planned was higher in the CRT group than the CTx group. The two groups were similar in terms of pT stage, histologic grade, tumor size, preoperative and postoperative CEA level, and perineural invasion.

**Fig 1:**
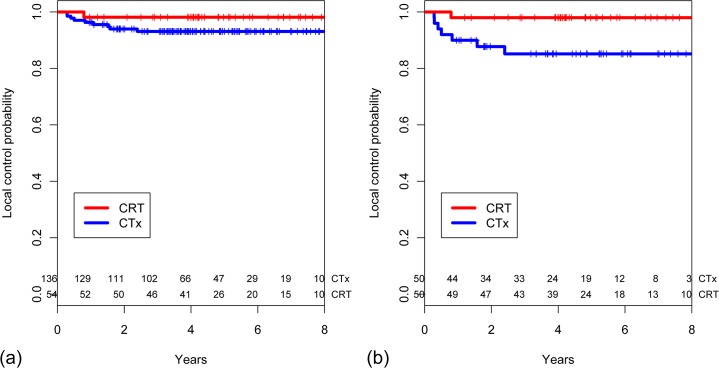
Estimated local control probability stratified based on receipt of postoperative chemoradiotherapy in (a) the entire cohort (n = 190) and (b) a propensity score-matched cohort (n = 100).

**Fig 2:**
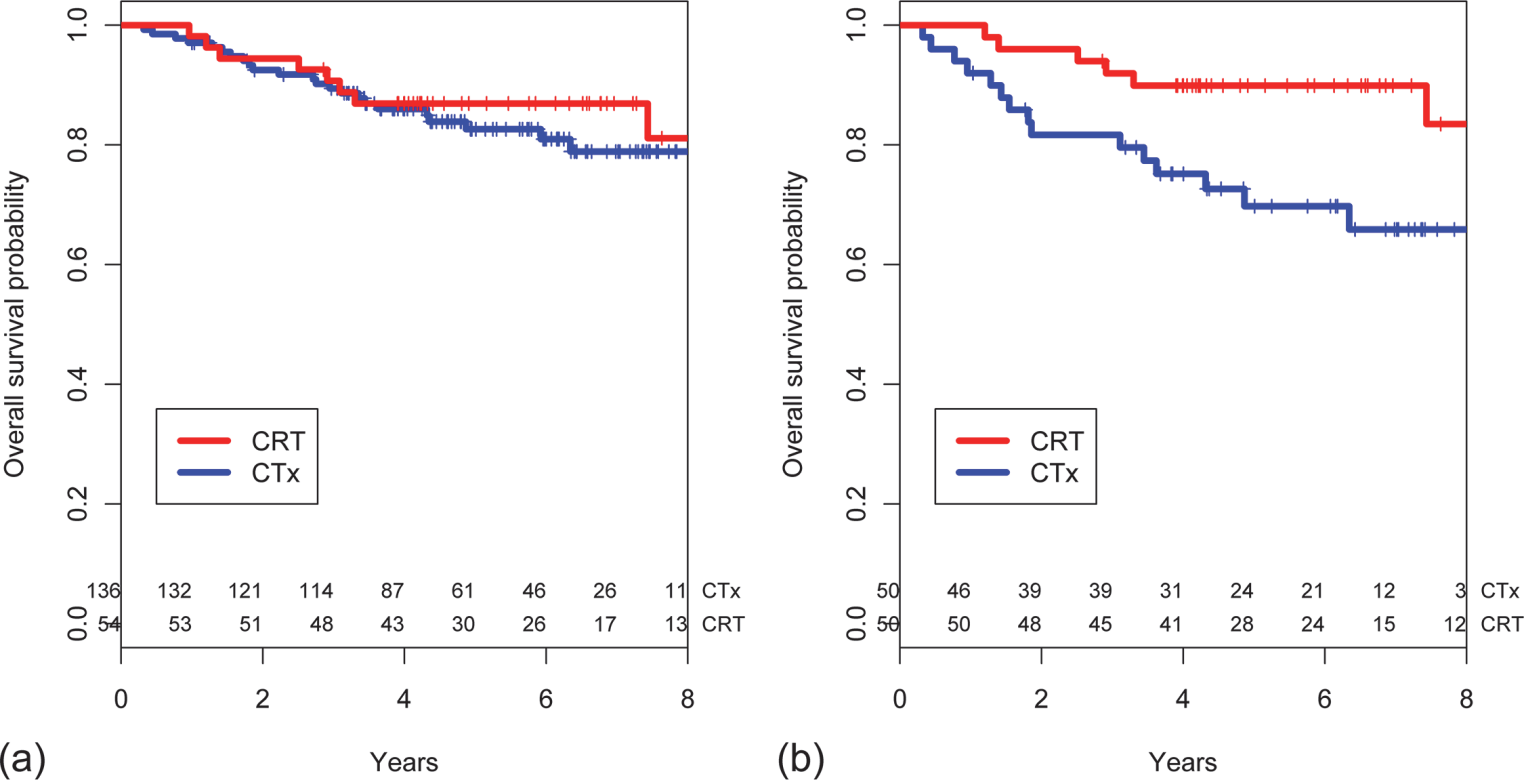
Estimated overall survival probability stratified based on receipt of postoperative chemoradiotherapy in (a) an entire cohort (n = 190) and (b) a propensity score-matched cohort (n = 100).

The median follow-up for survivors was 76 months (range 34–125 months) in the CRT group and 63 months (range 12–112 months) in the CTx group, respectively. There were no significant differences in outcomes between the two groups. The 5-year local control rates were 98.1% (95% confidence interval [95% CI] 87.6–99.7) in the CRT group and 93.1% (95% CI 87.2–96.4) in the CTx group (*p* = 0.183, Fig 1A). The 5-year OS rates were 86.9% (95% CI 74.5–93.5) and 82.6% (95% CI 74.4–88.4) in the CRT and CTx group, respectively (*p* = 0.689, Fig 2A). There were no differences between the CRT and CTx groups in terms of 5-year DMF (75.3% vs. 81.0%, *p* = 0.460) and RFS rates (73.6% vs. 76.0%, *p* = 0.576).

### Propensity score-matched cohort

Propensity score matching resulted in 50 matched pairs, for a total of 100 patients. Patient, tumor, and treatment characteristics were not significantly different between groups of the matched pairs (Table 1), indicating that the matching procedure worked well.

In the propensity score-matched cohort, the median follow-up for survivors was 61 months (range 36–77 months) in the CRT group and 63 months (range 34–81 months) in the CTx group. Receipt of CRT resulted in superior 5-year local control (98.0% [95% CI 86.6–99.7] vs. 85.2% [95% CI 71.2–92.7], *p* = 0.024, Fig 1B) and OS rate (89.9% [95% CI 77.4–95.7] vs. 69.8% [95% CI 54.0–81.0], *p* = 0.021, Fig 2B) compared with chemotherapy alone. The CRT group showed a trend towards better 5-year RFS (77.6% vs. 58.8%, *p* = 0.066) and DMF rates (79.5% vs. 62.8%, *p* = 0.060) compared with the CTx group.

### Subgroup analysis of all study patients

In a multivariate Cox proportional hazard analysis, receipt of CRT, pT stage, pN stage, histologic differentiation, and resection margin status were significantly associated with overall survival (S1 Table). To identify subpopulations of patients that benefit most from receiving CRT, subgroup analysis was performed by stratifying patients according to theses prognostic features. Of the 190 patients, 129 were considered low-risk (no poor prognostic features), and 61 as high-risk (having any of poor prognostic features including pT4, pN2, poor differentiation, and involved resection margin). Patient, tumor, and treatment characteristics of these patients according to risk factors are summarized in Table 2.

**Table 2:** Characteristics of low–risk patients (n = 129) and high–risk patients (n = 61), stratified by treatment type (CTx vs CRT).

Variable		Low–risk patients (n = 129)			High–risk patients (n = 61)		
		CTx (n = 103)	CRT (n = 26)	*P* value	CTx (n = 33)	CRT (n = 28)	*P* value
**Age**				0.014*			0.973
	**≤ 60 years**	36 (35.0)	16 (61.5)		14 (42.4)	12 (42.9)	
	**> 60 years**	67 (65.0)	10 (38.5)		19 (57.6)	16 (57.1)	
**Sex**				0.022*			0.441
	**Female**	62 (60.2)	22 (84.6)		18 (54.5)	18 (64.3)	
	**Male**	41 (39.8)	4 (15.4)		15 (45.5)	10 (35.7)	
**pT stage**				0.479			0.243
	**pT1/2**	10 (9.7)	4 (15.4)		3 (9.1)	0 (0)	
	**pT3/4**	93 (90.3)	22 (84.6)		30 (90.9)	28 (100)	
**pN stage**				0.448			0.493
	**pN0**	60 (58.3)	13 (50.0)		4 (12.1)	3 (10.7)	
	**pN1**	43 (41.7)	13 (50.0)		4 (12.1)	6 (21.4)	
	**pN2**	0 (0)	0 (0)		25 (75.8)	19 (67.9)	
**pTNM stage**				0.448			1.000
	**pStage II**	60 (58.3)	13 (50.0)		4 (12.1)	3 (10.7)	
	**pStage III**	43 (41.7)	13 (50.0)		29 (87.9)	25 (89.3)	
**Histologic grade**				–			0.974
	**Others**	0 (0)	0 (0)		27 (81.8)	23 (82.1)	
	**Poorly differentiated**	103 (100)	26 (100)		6 (18.2)	5 (17.9)	
**Tumor size (cm)**		4.5 (1.0–11.0)	3.9 (0.2–9.0)	0.612	5.5 (1.1–12.0)	5.5 (3.0–9.0)	0.796
**Number of harvested lymph nodes**		27 (7–89)	22 (12–39)	<0.001*	29 (13–119)	28 (12–76)	0.379
**Preoperative CEA (ng/mL)**		2.4 (0.9–49.7)	1.6 (0.9–13.0)	0.111	4.2 (0.9–215.0)	4.1 (0.9–40.0)	0.134
**Postoperative CEA (ng/mL)**		0.9 (0.3–11.2)	1.0 (0.5–4.5)	0.389	1.2 (0.8–7.8)	1.2 (0.5–4.4)	0.572
**Lymphovascular invasion**				0.547			0.544
	**No**	66 (64.1)	15 (57.7)		8 (24.2)	5 (17.9)	
	**Yes**	37 (35.9)	11 (42.3)		25 (75.8)	23 (82.1)	
**Perineural invasion**				0.161			0.554
	**No**	82 (79.6)	24 (92.3)		14 (42.4)	14 (50.0)	
	**Yes**	21 (20.4)	2 (7.7)		19 (57.6)	14 (50.0)	
**Involved resection margin**				–			0.037*
	**No**	103 (100)	26 (100)		25 (75.8)	14 (50.0)	
	**Yes**	0(0)	0(0)		8 (24.2)	14 (50.0)	
**Adjuvant chemotherapy for 6 months**				0.013*			1.000
	**No**	20 (19.4)	0 (0)		3 (9.1)	2 (7.1)	
	**Yes**	83 (80.6)	26 (100)		30 (90.9)	26 (92.9)	

Categorical variables are presented as n (%), continuous variables are presented as median (range).

*Abbreviations*: CTx = chemotherapy alone; CRT = chemoradiotherapy; CEA = carcinoembryonic antigen.

* Statistically significant.

**Fig 3:**
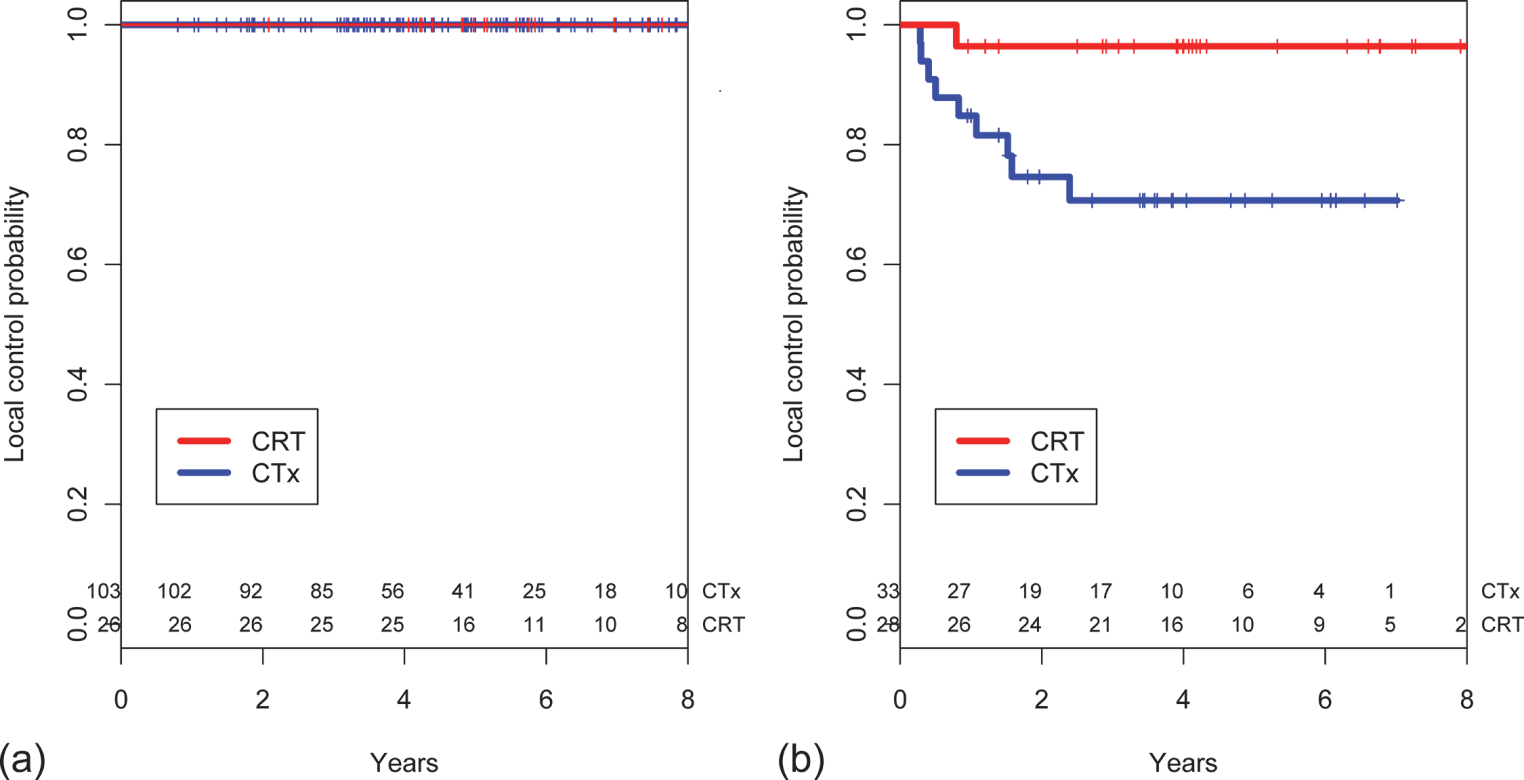
Estimated local control probability stratified based on receipt of postoperative chemoradiotherapy in (a) low-risk patients (n = 129) and (b) high-risk patients (n = 61).

**Fig 4:**
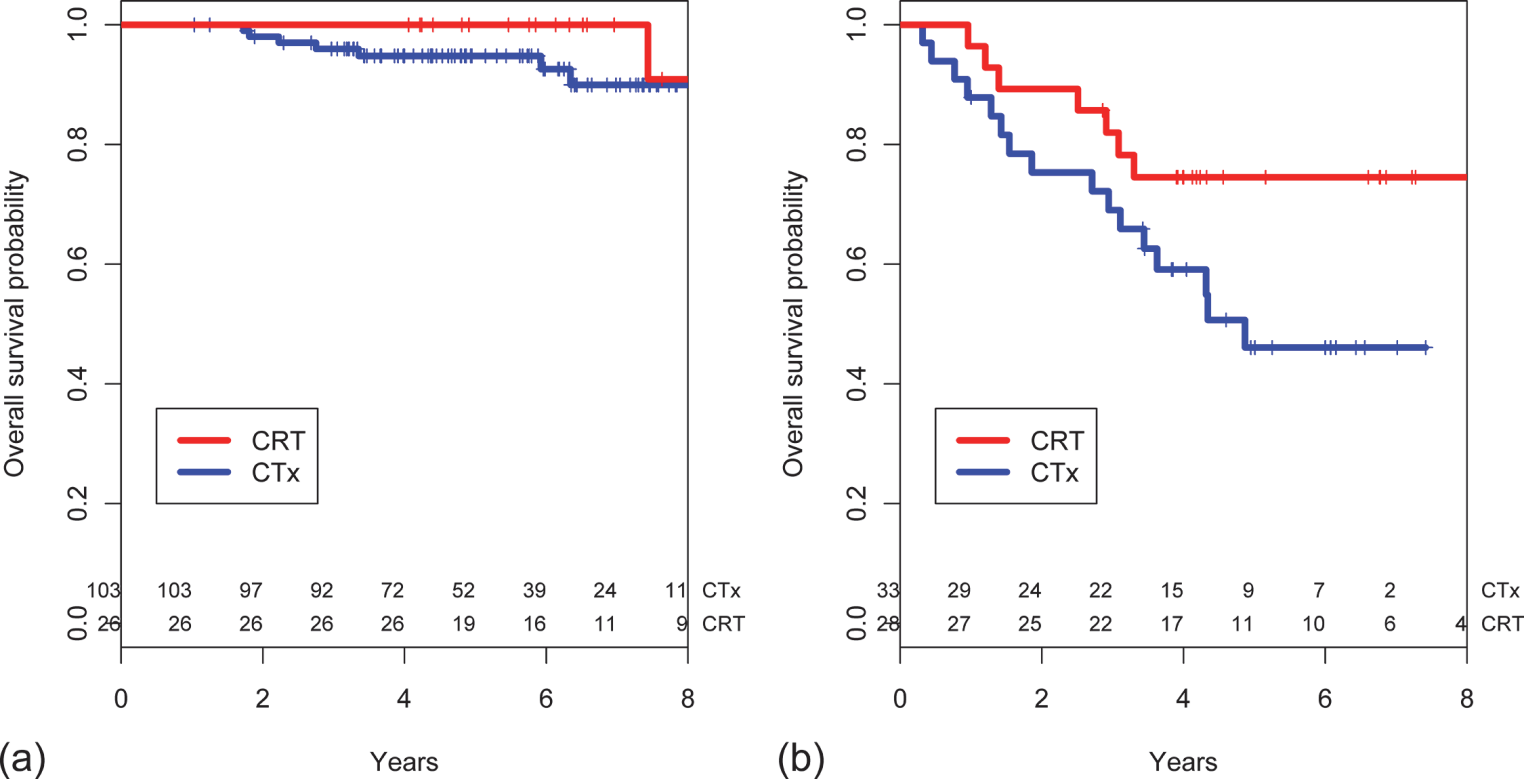
Estimated overall survival probability stratified based on receipt of postoperative chemoradiotherapy in (a) low-risk patients (n = 129) and (b) high-risk patients (n = 61).

CRT conferred no benefit in low-risk patients. Local recurrence did not occur in low-risk patients regardless of the receipt of CRT (Fig 3A). For high-risk patients, those who received CRT had a significantly higher 5-year local control rate than those who did not (96.4% [95% CI 77.2–99.5] vs. 70.7% [95% CI 50.9–83.7], *p* = 0.013, Fig 3B). CRT did not confer a survival benefit in low-risk patients (*p* = 0.712, Fig 4A). However, among high-risk patients a marginally positive effect of CRT on OS was observed. The 5-year OS rates were 74.5% (95% CI 53.8–87.0) and 46.1% (95% CI 27.0–63.2) in the CRT and CTx group, respectively (*p* = 0.074, Fig 4B).

## Discussion

The upper rectum is covered by peritoneum anteriorly and on both sides, while the mid rectum is only covered anteriorly and there is no peritoneal covering in the lower rectum. Consequently, when the transmural invasion depth of the primary tumor is similar, upper rectal tumors are more likely to penetrate the peritoneum. Tumors penetrating the peritoneum are more likely to disseminate within the peritoneal cavity and be beyond the field covered by pelvic radiotherapy [8]. For this reason, many oncologists hesitate to offer adjuvant radiation for upper rectal cancer. However, this perception was based on studies done in the 1980s. Due to the remarkable evolution of surgical, radiotherapeutic techniques, and chemotherapy, CRT should be re-evaluated in the context of contemporary oncologic treatments. In the current study, only two of 19 pT4 patients exhibited peritoneal seeding as the initial recurrence. Therefore, it could be postulated that locally advanced upper rectal cancer is rarely a systemic disease, suggesting that appropriately applied adjuvant pelvic radiotherapy may be beneficial.

Another reason adjuvant radiotherapy is not a standard treatment for locally advanced upper rectal cancer is that the benefit of adjuvant radiotherapy is not as clear as it is for mid and lower rectal cancer. The Dutch TME trial [6] and Swedish rectal cancer trial [7] demonstrated that, although local recurrence by preoperative radiotherapy in the mid and lower rectum was reduced significantly, no significant reduction in local recurrence was found in upper rectal cancer. However, the German rectal cancer study found decreased local recurrence with the use of adjuvant CRT in upper rectal cancer as in the mid and lower rectal cancer [5].

In the current study, we report outcomes of patients with stage II or III upper rectal cancer who were treated by the mainstay strategy of surgery followed by postoperative chemotherapy with or without the application of postoperative radiotherapy. Treatment outcomes of the current study were comparable with previous studies of upper rectal cancer [12–15], notwithstanding the high proportion of stage III disease (58%) included in the current study. According to a pooled analysis of three randomized rectal adjuvant studies, in which patients were given postoperative pelvic radiation alone or combined with concurrent adjuvant 5-FU-based chemotherapy [23], the 5-year cumulative incidence of rectal cancer local recurrence was 8% for T3N0 patients (n = 664), 6% for T1-2N1 patients (n = 225), and 11% for T3N1 patients (n = 536). The corresponding incidence rates of distant metastasis were 19%, 15%, and 34%, respectively. In contrast, in the current study, there was no local recurrence in T3N0/T1-3N1 patients (n = 139), and the 5-year cumulative incidence of distant metastasis was 9% for T3N0 patients (n = 76), 0% for T1-2N1 patients (n = 15), and 14% for T3N1 patients (n = 48). Although the results of these studies cannot be directly compared due to different patient compositions, our results suggest that upper rectal cancer is less likely to recur than mid and lower rectal cancer with selective use of radiotherapy. Furthermore, a large national population-based observational study of patients (n = 3196) also found that patients with mid and lower rectal cancer are more likely to have local recurrence in the setting of TME surgery without radiotherapy [19]. The Medical Research Council (MRC) CR07 study also reported a low local recurrence rate of 6.2% after postoperative CRT for upper rectal cancer and 9.8% and 10.4% for mid and lower rectal cancer, respectively [24]. Considering this relatively low incidence of recurrence of upper rectal cancer compared with mid and lower rectal cancer, it is highly desirable to select patients for additional radiotherapy who are most likely to benefit from it and consider omitting radiotherapy in patients with low risk factors. Striving for a more risk-adapted use of preoperative CRT in patients with stage II and III rectal cancer, some study groups attempted to demonstrate the prognostic impact of preoperative MRI assessment of CRM involvement [25, 26]. Although they insisted that the anticipated CRM assessed by MRI is superior to AJCC staging when choosing to undergo primary surgery or to receive preoperative CRT [25], the CRM was not predictive for local recurrence in multivariate analysis of the MRC CR07 study [27]. And in these MRI-based CRM studies [25, 26], adjuvant radiotherapy was not offered for patients with lymph node metastasis, while it is the most important independent risk factor for local recurrence [27–29]. It is doubtful whether the omission of radiotherapy can be compensated by chemotherapy alone in patients with lymph node metastasis. Furthermore, according to a methodological analysis of studies on the accuracy of preoperative MRI to predict negative from positive CRM in the specimen, the authors concluded that “MRI cannot predict tumor involvement of a CRM” [30]. In the current study, we found T stage, N stage, histologic differentiation, and resection margin as poor prognostic factors. These factors may need to be considered to omit radiotherapy.

There were several limitations to our study. First, due to the relatively small sample size of patients who received postoperative CRT, despite being one of the largest studies of upper rectal cancer to be reported, the survival benefit of CRT in high-risk patients (n = 61) did not reach statistical significance. Nevertheless, among the 28 patients who received postoperative CRT, only 7 (25.0%) patients died during the follow-up period, while among the 33 patients who did not receive postoperative CRT, 16 (48.5%) patients died. Second, while we have tried to adjust for possible confounders by using propensity score matching and stratification, unknown or unmeasured confounders may still persist, highlighting the need for large-scale prospective randomized trials to confirm the findings.

## Conclusion

After adjusting for clinicopathologic factors by propensity score matching, postoperative CRT was associated with improved local control and overall survival in stage II and III upper rectal cancer. And we observed that stage II and III upper rectal cancer is less likely to recur without routine use of preoperative CRT. Our results suggest that surgery followed by chemotherapy alone is acceptable for patients who did not have poor prognostic features, while additional radiotherapy should be given for patients who have any poor prognostic features (pT4, pN2, poor differentiation, and involved resection margin).

## Supporting Information

S1 Dataset Original clean data for analysis. (XLSX)

S1 Table Multivariate analysis to identify prognostic factors for overall survival. (DOCX)
