# Supplementary material for: Impact of Postoperative Chemoradiotherapy versus Chemotherapy Alone on Recurrence and Survival in Patients with Stage II and III Upper Rectal Cancer: A Propensity Score-Matched Analysis
Source: PLoS One. 2015 Apr 22;10(4):e0123657. doi: 10.1371/journal.pone.0123657 (PMC4406553; doi:10.1371/journal.pone.0123657)
Supplement: S1 Table — (DOCX) [file pone.0123657.s002.docx]

| **Variable** |  | **HR** | **95% CI for HR** | ***P* value** |
| --- | --- | --- | --- | --- |
| **Age** |  |  |  |  |
|  | **≤ 60 years** | 1 | – | – |
|  | **> 60 years** | 1.70 | 0.77–3.77 | 0.192 |
| **pT stage** |  |  |  |  |
|  | **pT1**–**3** | 1 | – | – |
|  | **pT4** | 2.72 | 1.16–6.36 | 0.021***** |
| **pN stage** |  |  |  |  |
|  | **pN0** | 1 | – | – |
|  | **pN1** | 1.51 | 0.48–4.75 | 0.483 |
|  | **pN2** | 5.50 | 1.80–16.71 | 0.003***** |
| **Histologic grade** |  |  |  |  |
|  | **Others** | 1 | – | – |
|  | **Poorly differentiated** | 3.96 | 1.58–9.93 | 0.003***** |
| **Involved resection margin** |  |  |  |  |
|  | **No** | 1 | – | – |
|  | **Yes** | 3.91 | 1.59–9.62 | 0.003***** |
| **Receipt of PORT** |  |  |  |  |
|  | **No** | 1 | – | – |
|  | **Yes** | 0.40 | 0.15–0.99 | 0.048***** |

**Table S1. Multivariate analysis to identify prognostic factors for overall survival**

Abbreviations: HR = hazard ratios; CI = confidence intervals; PORT = postoperative chemoradiotherapy.

* Statistically significant.
